# Community-based health insurance dropout rate and its' associated factors among households in Seyo District, Kellem Wollega Zone, Oromia, Ethiopia: community-based cross-sectional study

**DOI:** 10.3389/fpubh.2025.1562257

**Published:** 2025-06-12

**Authors:** Segni Mulugeta Tafasa, Yonas Etana, Firezer Belay Keno, Worku Fikadu, Dejene Seyoum

**Affiliations:** ^1^School of Public Health, Institute of Health Science, Wallaga University, Nekemte, Ethiopia; ^2^Department of Public Health, Institute of Health Science, Dambi Dollo University, Dambi Dollo, Ethiopia; ^3^Department of Public Health, College of Medicine and Health Sciences, Arsi University, Asella, Ethiopia

**Keywords:** Community-based health insurance, dropout, factor associated, Seyo, Dropout rat

## Abstract

**Background:**

Despite an initial surge in enrollment in the Community-Based Health Insurance (CBHI) scheme, maintaining the membership remains a significant challenge in Ethiopia, particularly in the study area. High dropout rates jeopardize the program's effectiveness and long-term sustainability. Therefore, this study aimed to assess the CBHI dropout rate and its associated factors among households in Seyo District, Kellem Wollega, Ethiopia.

**Methods:**

A community-based cross-sectional study was conducted among 551 randomly selected households from eight kebeles in the Seyo district. This study employed a multistage sampling technique and took place from 1 August to 30 September 2023. Data were collected using pretested and structured questionnaires. The collected data were coded, entered into Epi Info (version 7), and analyzed using Statistical Package for the Social Sciences (SPSS version 25). Descriptive statistics were computed for key variables. Bi-variable and multivariable logistic regression analyses were performed to identify factors associated with CBHI dropout. Adjusted odds ratios (AORs) with 95% confidence intervals (CIs) were calculated to assess the strength of these associations. A *p*-value of < 0.05 was considered statistically significant. The results are presented as tables and graphs.

**Results:**

A total of 546 households participated in the study, yielding a response rate of 99.1%. The overall CBHI dropout rate was 36.1% (95% CI, 32–40%). Factors significantly associated with the CBHI dropout rate included family size (AOR = 1.94, 95% CI: 1.18–3.19), age ≥51 years (AOR = 0.39, 95% CI: 0.18–0.86), being unable to read and write (AOR = 5.58, 95% CI: 2.88–10.8), being able to read and write (AOR = 3.59, 95% CI: 1.76–7.33), attending primary education (AOR = 2.45, 95% CI: 1.25–4.81), being a paying member (AOR = 2.52, 95% CI: 1.31–4.86), no history of chronic illness in the family (AOR = 1.75, 95% CI: 1.09–2.8), leaving health facilities without receiving treatment (AOR = 3.9, 95% CI: 2.29–6.57), and insufficient or unavailable laboratory services (AOR = 1.95, 95% CI: 1.15–3.32).

**Conclusion:**

More than one-third of the households in the study area dropped out of the CBHI scheme. This study identified several factors that are significantly associated with the CBHI dropout rate. These factors include family size, age of the household head, educational level, type of membership, absence of chronic illness in the household, experiences of leaving health facilities without treatment, and lack of adequate laboratory services. Therefore, we strongly recommend that the government and relevant stakeholders take action to address these factors in order to reduce dropout rates and improve the sustainability of the scheme.

## Introduction

Universal Health Coverage (UHC) is a global priority that emphasizes the need to improve financial access to healthcare services, protect populations from catastrophic health expenditures, and reduce the risk of extreme poverty through effective health insurance schemes. Ethiopia's healthcare system has made notable progress over the past two decades, primarily due to increased public investment, health extension programs, and the expansion of primary healthcare units. However, the healthcare system remains underfunded and heavily dependent on out-of-pocket (OOP) payments, which limits access to care, particularly for rural and low-income populations. According to the Ethiopian Ministry of Health (2023), OOP expenditure still accounts for over 30% of total health spending, resulting in catastrophic health expenditures for millions of people. In response to these financial barriers, Ethiopia introduced the Community-Based Health Insurance (CBHI) scheme to enhance equity in health financing, increase service utilization, and protect households from impoverishment due to healthcare costs (1, 2).

The CBHI scheme was initially launched in 2011 across 13 woredas in four regions (Tigray, Amhara, Oromia, and SNNPR), targeting informal sector workers and rural communities. Following the pilot program's success, the scheme was gradually expanded nationwide. In the Wollega Zone of the Oromia Region, the implementation of the CBHI scheme began in phases after 2013, in line with the federal expansion strategy. The scheme aimed to address local challenges such as low service utilization and high OOP expenditures, particularly in rural kebeles where access to health services is limited (3).

To expand prepaid health coverage and improve access to modern healthcare services, the Ethiopian government has developed a comprehensive health insurance strategy. Two insurance schemes were introduced: Social Health Insurance (SHI) and Community-Based Health Insurance (CBHI). SHI, which is currently in the implementation phase, intended to cover ~10.46% of the formal sector workforce. Community-Based Health Insurance (CBHI) is a voluntary insurance model organized at the community level, often referred to as mutual health organizations or micro-insurance schemes. CBHI provides financial protection by reducing OOP expenditures and improving cost recovery (2).

Governments in low- and middle-income countries (LMICs) have adopted these schemes to achieve UHC by enhancing healthcare access, utilization, and financial protection, primarily by reducing direct OOP payments. CBHI schemes have been operational in sub-Saharan Africa since the 1990s (4). These schemes aim to ensure that sufficient resources are available to enable members to access quality healthcare. In Ethiopia, the government has used CBHI to alleviate OOP burdens by enrolling informal sector workers from both rural and urban communities (5).

Globally, ~25 million households (equivalent to 100 million people) are pushed into deep poverty each year due to OOP healthcare expenses, particularly in LMICs. These challenges are often exacerbated by man-made and natural disasters, which further increase healthcare costs at individual, familial, and national levels. Consequently, healthcare financing remains a pressing global issue. Reports show that OOP expenditure accounts for 38.5% of healthcare spending in high-income countries, 63%−86% in Southeast Asia, and over 40% in Africa (6, 7).

To address financial challenges and promote healthcare-seeking behavior, the Ethiopian government launched the CBHI initiative. Although initial enrollment is essential, sustainability depends on continued renewal of memberships. However, high dropout rates remain a persistent issue in many LMICs. For example, a report from Asia found that ~80% of enrolled members dropped out of CBHI programs (8). In the Gujarat and Maharashtra districts of India, dropout rates were 49 and 67%, respectively (9, 10). In Africa, dropout rates range from 6.8 to 83% (11–14). In most sub-Saharan African countries, with the exceptions of Ghana and Rwanda, CBHI membership rates remain below 10% (15, 16).

In Ethiopia, a limited number of studies have reported high CBHI dropout rates. For instance, research conducted in Gumbichu (North Shoa) and West Shoa reported dropout rates of 74.7 and 38%, respectively (17, 18). Other studies in Dera (North Shoa) and Manna (Jimma Zone) recorded dropout rates of 37.3 and 31.9%, respectively (19, 20).

Various studies globally have identified several factors associated with high CBHI dropout rates (17–22). These factors include a lack of understanding of health insurance, prior experience with CBHI schemes, limited knowledge of benefits, accessibility of health facilities, healthcare needs, quality of care, household demographics, long waiting times, unaffordable premiums, inconvenient payment models, female-headed households, older household heads, low educational status, fewer disease episodes or dependents among household members, rigid scheme regulations, inadequate legal and policy frameworks, and inappropriate benefit packages (17–19, 21, 23–30).

To reduce the dropout rate from the CBHI scheme, the Ethiopian government has implemented various strategies, including third-party reimbursement and contingency funds, to support the poorest households. Despite these interventions, dropout rates from the CBHI scheme continue to be a significant challenge across the country—particularly in the study area. Therefore, this study aimed to assess the CBHI dropout rate and its associated factors among households in the Seyo district.

## Methods and materials

### Study area and period

The study was conducted in the Seyo district, which is located in the Kellem Wollega Zone of the Oromia Regional State, ~652 km west of Addis Ababa, the capital city of Ethiopia. According to 2022–2023 governmental estimates, the district has a total population of 112,190, composed of 56,881 female and 55,309 male individuals. The district consists of 27 kebeles, five government health centers, and 26 health posts that provide health services to the community. The study was carried out from 1 August 2023, to 30 September 2023.

### Study design

A community-based cross-sectional study design was employed.

### Population

The source population for the study consisted of all households that were previously enrolled in the CBHI scheme within the district, while the selected kebeles served as the study population. Household heads and their spouses who had ever been enrolled in the CBHI scheme within the selected kebeles were included in the study. However, household heads who held CBHI memberships but had relocated from other areas, as well as those who were newly enrolled during the data collection period, were excluded from the study.

### Sample size determination and sampling procedures

#### Sample size determination

The sample size was calculated using both single and double population proportion formulas for the first and second objectives, respectively. For the first objective, the calculation was based on an assumed dropout rate of 31.9% from the Community-Based Health Insurance scheme, which was obtained from a community-based cross-sectional study conducted in the Manna district (20). A 95% confidence level, a 5% margin of error, and a design effect of 1.5 were taken into account during the calculation.


(1) $$\begin{array}{l} n=\frac{{\left({z\alpha /}_{2}\right)}^{2}\times pq}{d^{2}}=n=\frac{{\left(1.96\right)}^{2}\times 0.319\times \left(1-0.319\right)}{{\left(0.05\right)}^{2}}=334 \end{array}$$

➢ *n* = sample size
➢ *z* = Confidence level [95%]
➢ *d* = margin of error at 0.05
➢ *p* = proportion of households dropping out from the CBHI scheme
➢ *q* = 1 – *p*.
  Finally, after multiplying by a design effect of 1.5 and accounting for a non-response rate of 10%, the final sample size for the first objective was determined to be 551.

For the second objective, the sample size was determined using an assumption of 80% power and 95% CI. This calculation was performed using Epi Info StatCalc, focusing on the most significant variable from the previous study (Table 1).

**Table 1:** Sample size calculation for second objective.

**S/N**	**Selected variable**	**Prevalence among exposed**	**Prevalence among the unexposed**	**Sample size**	**By adding a 10% non-response rate and multiplying by a 1.5 design effect**
1	Household size (18)	Magnitude of DO among family sizes of >5 = 58.3% AOR = 0.36	Magnitude of DO among family sizes of < 5 = 41.7%	308	508
2	Presence of chronic illness (18)	Magnitude of DO among families with chronic illness = 68.5% AOR= 0.159	Magnitude of DO among families without chronic illness = 31.5%	66	198
3	Knowledge of participants on the CBHI scheme (19)	Magnitude of DO among participants with good knowledge = 25%	Magnitude of DO among participants with poor knowledge = 12.3%	324	535

Among the two objectives, the sample size calculated for the first objective is the highest, totaling 551 participants.

#### Sampling procedure

**Figure 1:**
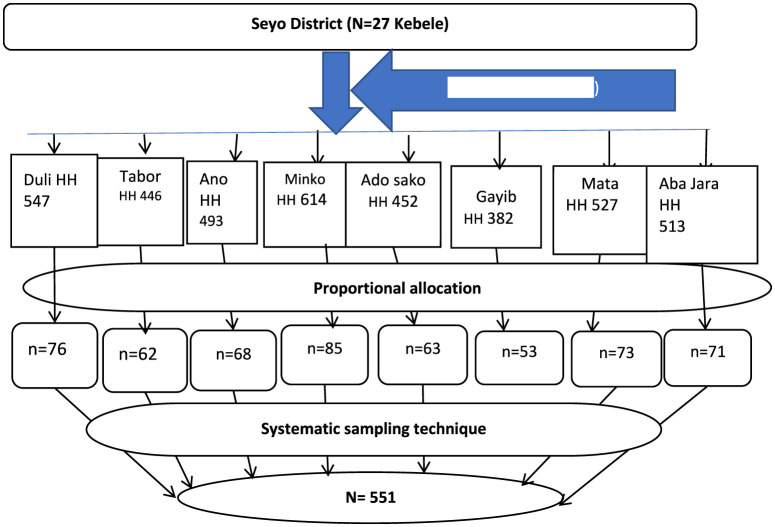
Diagrammatic representation of sampling procedures in Seyo District, Kellem Wollega, Oromia, Ethiopia, in 2023. HH, household; SRS, simple random sampling.

A multistage sampling technique was employed to select the study participants. In the first stage, eight kebeles were randomly selected from the 27 kebeles in the Seyo district using a lottery method. In the second stage, the sample size was proportionally allocated to each selected kebele based on the size of their respective households. Then, the study participants within each selected kebele were selected through systematic sampling, utilizing household enrollment identification numbers from the CBHI registration book as the sampling frame (Figure 1).

### Study variables

#### Dependent variable

The dropout rate from the Community-Based Health Insurance scheme is the dependent variable.

#### Independent variables

➢ Sociodemographic and economic factors: age of household (HH) head, sex of HH head, educational level, household size, and distance from the health facility.
➢ Health and Health-related factors: chronic illness, availability of health services, illness episodes in the past 3 months, health-seeking behavior, waiting time, and perceived quality of healthcare services.
➢ CBHI-related factors: customer information about CBHI, package benefits, affordability, scheme experience, premium collection convenience, and provider's attitude.
➢ Individual- and household-related factors: length of enrollment, attitude toward CBHI, CBHI knowledge, understanding of CBHI, trust in health facilities, trust in CBHI schemes, and frequency of health facility visits.

### Operational definitions

Dropout: Households that did not renew their membership after a year (HHs that discontinued their membership) were considered to have dropped out, while those that did renew were not classified as having dropped out (18, 21, 22).

Dropout rate: It refers to the percentage or proportion of members who have dropped out in relation to the total number of individuals who were previously enrolled.

Renewal: “Renewal” includes households that had CBHI for more than 1 year and were still enrolled at the time of the survey. It also applies to households that were enrolled in the first year of operation, dropped out in the second year, and enrolled again in the third year.

Membership: Households join the CBHI scheme by paying the preset contribution and receive an identification card for the scheme.

### Data collection tools and procedures

Data were collected using interviewer-administered, pretested, and structured questionnaires adapted from related studies (18, 20–22). The questionnaire consisted of three parts: the first part covered sociodemographic characteristics, the second focused on CBHI utilization, and the third addressed health and health-related variables. It was initially prepared in English, translated into Afan Oromo, and then back-translated into English by a language expert to ensure consistency. Data collection was carried out by six nurses with diploma-level qualifications, and it was supervised by two public health professionals.

### Pretest of the questionnaire

A pretest was conducted to assess the clarity, relevance, and consistency of the questionnaire before the actual data collection. The pretest was conducted in Hawa Galan, a neighboring woreda with sociodemographic characteristics similar to the study setting, before the actual study began. Approximately 5% of the calculated sample size (29 households) participated in the pretest. The pretest identified unclear or ambiguous questions, assessed the logical flow of the questionnaire, and evaluated the appropriateness of the terminology used.

### Data quality control

To ensure the quality of the data, a 1-day orientation was provided to data collectors and supervisors by the principal investigator. The training focused on how to accurately complete the questionnaire. All collected data were checked daily by the supervisors for completeness and consistency. On-site supervision and technical support were provided throughout the data collection process by both the supervisors and the principal investigator. A pretest was conducted on 5% of the sample size in a neighboring district before the actual data collection began.

### Data processing and analysis

After data collection, the data were checked for completeness, entered into Epi Info (version 7), and analyzed using SPSS (version 25). Data cleaning was conducted before the analysis. Descriptive statistics, including frequencies, cross-tabulations, and summary measures, were computed for relevant variables. The normality of continuous variables was checked and found to be normally distributed. Multicollinearity between independent variables was assessed using the Variance Inflation Factor (VIF), and no multicollinearity issues were identified.

Bivariable logistic regression analysis was performed to identify candidate variables for multivariable logistic regression. Variables with a *p*-value of < 0.25 in the bivariable analysis were included in the multivariable logistic regression model. Model fitness was assessed using the Hosmer-Lemeshow goodness-of-fit test. Adjusted odds ratios (AORs) with 95% confidence intervals (CIs) were used to assess the strength of associations, and statistical significance was declared at *p* < 0.05.

### Ethical Considerations

The study proposal was submitted to the Department of Public Health, Institute of Health Sciences, Dambi Dollo University. After a detailed review, the Research Ethics Committee (REC) of the institute provided an ethical approval letter. Upon approval, a letter of permission was obtained and submitted to the relevant authorities. The district health office issued supportive letters to each kebele. Informed consent was obtained from all participants. Confidentiality and privacy of the information were strictly maintained. Participants were informed that their participation was voluntary and that they had the right to withdraw at any time.

## Results

### Sociodemographic and economic characteristics of the respondents

A total of 546 participants participated, with a response rate of 99.1%. The mean age of respondents was 45 years (45 ± 10.82 SD). The majority of the respondents, 500 (91.6%), were men, and 515 (94.3%) were married. Approximately 178 (32.6%) participants could not read or write. The majority of respondents, 536 (98.2%), lived in rural areas. Additionally, 188 (34.4%) respondents were over 51 years old. Overall, 87.5% of the participants were farmers. Approximately 375 (68.7%) households had fewer than five family members (see Table 2).

**Table 2:** Sociodemographic characteristics among households in the Seyo district, Kellem Wollega zone, Oromia, Ethiopia, in 2023.

**Variables**	**Category**	**Frequency**	**Percent (%)**
Residence	Rural	536	98.2
	Urban	10	1.8
Sex of respondent	Male	500	91.6
	Female	46	8.4
Ethnicity	Oromo	523	95.8
	Amara	23	4.2
Religion	Protestant	320	58.6
	Muslim	127	23.3
	Orthodox	99	18.1
Marital status	Married	515	94.3
	Divorced	11	2
	Widowed	20	3.7
Educational status	Unable to read and write	178	32.6
	Only able to read and write	106	19.4
	Primary education	158	28.9
	Secondary education and above	104	19
Occupation	Farmer	478	87.5
	Merchant	44	8.1
	Daily laborer	24	4.4
≥65 years old in the HHs	Yes	185	33.9
	No	361	66.1
Number of PW in the HHs	0	317	58.1
	≥1	229	41.9
Age	18–30	55	10.1
	31–40	149	27.3
	41–50	154	28.2
	≥51	188	34.4
Family size	< 5	375	68.7
	≥5	171	31.3

PW, Pregnant women.

### Community-Based Health Insurance (CBHI) scheme status of households

Among the study participants, 457 (83.7%) households were paying members, while 89 (16.3%) were among the poorest and were exempt from contributing to the CBHI program. Almost all participants, 485 (88.8%), possessed CBHI identification cards. Of those who renewed their membership, only 19 individuals (5%) consistently maintained their membership over the past 5 years.

Out of 546 respondents, 197 (36.1%) (36.1%, 95% CI: 32%−40%) canceled their membership, while 349 (63.9%) renewed it. Those who did not renew their membership cited various reasons, including low quality of healthcare services (91 respondents, 46%) and lack of awareness about how CBHI works (35 respondents, 18%).

Regarding the sources of information about CBHI, ~348 (63.7%) received information from officials during public meetings, followed by 166 (30.4%) who were informed by health professionals at health facilities. The main reasons for initial enrollment in the CBHI scheme included exemption from registration fees and premiums (365 respondents, 66.8%) and the relatively low premium compared to user fees (89 respondents, 16.3%; see Table 3).

**Table 3:** Community-based health insurance status-related information among households in the Seyo district, Kellem Wollega zone, Oromia, Ethiopia, in 2023.

**Variables**	**Category**	**Frequency**	**Percent (%)**
Have CBHI ID	Yes	485	88.8
	No	61	11.2
Membership type	Payer	457	83.7
	Indigent	89	16.3
Source of CBHI information	CBHI officials in a public meeting	348	63.7
	Health professional in a health facility	166	30.4
	CBHI house-to-house awareness creation	22	4
	other source	10	1.8
Reason for having membership	Illness and/or injury occur frequently	365	66.8
	Pregnant women in the household	89	16.3
	Child/children in the household	65	11.9
	To finance health care expenses	11	2
	Households are exempt from the registration fee and premium	8	1.5
	Premium is low compared to the user	7	1.3
Dropout from the CBHI scheme	No	349	63.9
	Yes	197	36.1
Reason for renewing CBHI membership	Premium is low compared to the user fee	240	44
	Child/children in our HH needed	73	13.4
	Illness and/or injury occur frequently	13	2.4
	To finance unexpected health care expenses	9	1.6
	Pressure from the CBHI official	8	1.5
	Pregnant women in our HH	6	1.1
Do you have an intention to renew again when your current membership expires	Yes	349	63.9
	No	197	36.1
Single reason for not renewing	The quality of health care services is low	91	16.7
	Lack of awareness about the details of how the CBHI works	35	6.4
	The benefit package does not meet our needs	28	5.1
	CBHI management staff is not trustworthy	13	2.4
	The registration fee and premiums are not affordable	10	1.8
	Did not use services last year	8	1.5
	Want to wait in order to confirm the benefits of the scheme from others	6	1.1
	Illness and/or injury do not occur frequently in our HH	6	1.1

### Factors associated with the dropout rate from the CBHI scheme

In multivariate analysis, several factors such as family size, age of the respondent, educational status, type of membership, history of chronic illness, leaving a health facility without receiving treatment, and lack of sufficient and necessary laboratory services were significantly associated with the CBHI dropout rate.

This study showed that households with a family size of less than five were 1.94 times more likely to drop out from the CBHI scheme than those with a family size of five or more [AOR = 1.94 (1.18–3.19)]. The age of the household head was significantly associated with dropouts from the CBHI program. Accordingly, the likelihood of dropping out from the CBHI scheme decreased by 61% among household heads in age groups ≥51 years compared to household heads in age groups of 18–30 years [AOR = 0.39 (0.18–0.86)]. In terms of the educational status of household heads, individuals who were unable to read and write, able to read and write, and attended primary education were 5.58 [AOR = 5.58 (2.88–10.8)], 3.59 [AOR = 3.59 (1.76–7.33)], and 2.45 [AOR = 2.45 (1.25–4.81)] times more likely to drop out from the CBHI scheme than those who attended secondary and higher education, respectively.

The likelihood of dropping out from the CBHI scheme was higher among payer members compared to indigent members [AOR = 2.52 (1.31–4.86)]. The absence of a history of chronic illness was strongly associated with dropout from the CBHI scheme. A household with no history of chronic illness was almost twice as likely to drop out from the CBHI scheme when compared to its counterpart [AOR = 1.75 (1.09–2.8)].

A household that left a public health facility without receiving treatment was four times more likely to drop out of the CBHI membership compared to those that received treatment [AOR = 3.9 (2.29–6.57)]. The lack of sufficient and necessary laboratory services at public health facilities was significantly associated with the CBHI dropout rate. Study participants who visited health facilities without sufficient and necessary services were twice as likely to drop out from the CBHI scheme compared to those who visited health facilities with sufficient and necessary laboratory services [AOR = 1.95 (95% CI: 1.15%−3.32%); Table 4].

**Table 4:** Bivariate and multivariate results of factors associated with CBHI dropout among households in Seyo District, Kellem Wollega, in 2023.

**Variables**		**Dropou**		**COR (95% CI)**	**AOR (95% CI)**	***p*-Value**
		**No**	**Yes**			
Residence	Rural	346	190	1		
	Urban	3	7	4.25 (1.1–16.6)	4.2 (.8–22.6)	0.098
Marital status	Married	337	178	1		
	Divorced	6	5	1.6 (0.5–5.2)	0.99 (0.26–3.8)	0.99
	Widowed	6	14	4.4 (1.7–11.7)	1.73 (0.54–5.59)	0.36
Family size	< 5	220	155	1	1	
	≥5	129	42	2.2 (1.4–3.2)	1.94 (1.18–3.19)	0.009
Age	18–30	30	25	1	1	
	31–40	96	53	0.7 (0.4–1.2)	0.53 (0.25–1.12)	0.097
	41–50	92	62	0.8 (0.4–1.5)	0.59 (0.27–1.26)	0.173
	≥51	131	57	0.5 (0.3–0.97)	0.39 (0.18–0.86)	0.019
Education level	Unable to read and write	89	89	3.5 (2.04–6.09)	5.58 (2.88–10.8)	0.001
	Only able to read and write	66	40	2.1 (1.16–3.92)	3.59 (1.76–7.33)	0.001
	Primary education	113	45	1.4 (0.79–2.5)	2.45 (1.25–4.81)	0.009
	Secondary education and above	81	23		1	
Occupation	Farmer	308	170	1
	Merchant	24	20	1.5 (0.8–2.8)	1.58 (0.73–3.43)	0.251
	Daily laborer	17	7	0.7 (0.3–1.8)	0.57 (0.19–1.75)	0.314
Membership type	Payer	275	182	3.3 (1.82–5.87)	2.52 (1.31–4.86)	0.006
	Indigent	74	15	1		
Attending CBHI-related meetings	Yes	201	86	1		
	No	148	111	1.8 (1.2–2.5)	1.75 (0.99–3.07)	0.053
Frequency of meeting participation	≤ 1	221	136	1.3 (0,9–1.9)	1.2 (0.68–2.13)	0.531
	≥2	128	61	1		
Attending CBHI management	Yes	111	51	1		
	No	238	146	1.4 (0.9–2)	1.2 (0.72–2)	0.487
Having an official position in the kebele	Yes	83	36	1		
	No	266	161	1.4 (0.9–2.2)	0.97 (0.54–1.75)	0.925
Having a history of chronic disease	Yes	88	74	1.8 (1.2–2.6)	1.75 (1.09–2.8)	0.021
	No	261	123	1		
History of illness in the HH during the last 12 months	Yes	88	74	1		
	No	261	123	1.9 (1.2–2.8)	1.31 (0.74–2.35)	0.355
Distance from HF	≤ 5 km	313	162	1		
	>5 km	36	35	1.9 (1.2–3.1)	1.53 (0.83–2.84)	0.172
Leaving public HF without treatment	Yes	169	48	2.9 (2–4.3)	3.9 (2.29–6.57)	0.001
	No	180	149	1		
Availability of laboratory services in the visited HF	Yes	255	122	1		
	No	94	75	1.7 (1.1–2.4)	1.95 (1.15–3.32)	0.014

## Discussion

This study assessed the magnitude of the CBHI dropout rate and its associated factors among households in Seyo district, Kellem Wollega zone, Oromia, Ethiopia. The study found a CBHI dropout rate of 36.1% among the households in the study area. This finding is consistent with the results from Burkina Faso (31–46%), West Shoa (38%), and the Dera district (37.3%) (13, 18, 19).

However, the current CBHI dropout rate was lower than those reported in Senegal (72.6%) and Gumbichu (74.7%). This variation may be attributed to differences in study settings, socioeconomic and cultural backgrounds, study periods, and sample sizes (14, 17).

Conversely, our finding indicated a higher dropout rate compared to studies conducted in Uganda and Vietnam, which reported CBHI dropout rates of 25.1 and 21.2%, respectively (24, 28). This discrepancy could be due to differences in the study periods, socioeconomic status, health-seeking behaviors, and national health policies. Similarly, our results surpassed the 31.9% dropout rate reported in the Manna district of the Jimma zone. This discrepancy may be attributed to the difference in the implementation of the CBHI scheme. Another possible reason may be differences in community awareness (20).

Households with smaller family sizes were more likely to drop out of the CBHI scheme than their counterparts. This finding aligns with studies conducted in rural India and the Manna district of the Jimma zone (8, 20). This may be because larger families are more likely to experience illness, increasing the risk of medical expenses, which motivates them to stay enrolled in the CBHI program to mitigate financial risks.

Household heads aged 51 years and above were less likely to drop out than those aged 18–30 years. This is consistent with studies conducted in Vietnam, the Manna district, and the West Shewa zone of the Oromia region (18, 20, 28). This is because older individuals may have decreased immunity, making them more prone to illness and thus more inclined to remain enrolled. Additionally, with age comes experience anticipating financial hardships, particularly related to healthcare, prompting continued participation in the CBHI scheme.

Regarding educational status, household heads who were unable to read and write or had only basic education (able to read and write or attended primary school) were more likely to drop out compared to those with secondary or higher education. This finding aligns with the studies conducted in Sudan, Bangladesh, and Manna district (20, 31, 32). One possible explanation is that more educated individuals are more likely to understand the benefits of health insurance and are generally more receptive to health reforms. They also tend to be more aware of the potential health crises and the importance of risk mitigation.

Payer members were more likely to drop out compared to indigent members. This aligns with the findings from the Gurage Zone in Southern Ethiopia (22). As payments for indigent members are covered by a third party, they may be more inclined to remain in the scheme without financial concerns.

Households without a history of chronic illness were nearly twice as likely to drop out compared to those with a history of chronic illness. This is consistent with the findings from West Shoa, Northwest Ethiopia, and Karnataka, India (18, 21, 33). A possible reason for this is that households without chronic illnesses may not visit healthcare facilities frequently, reducing their perceived need for insurance coverage.

Households that left public health facilities without receiving treatment were more likely to drop out from the CBHI scheme than those who received services. This finding is in line with studies conducted in rural districts of the Gurage zone (22). The availability of essential supplies, such as medications, plays a crucial role in encouraging continued participation in the CBHI scheme.

Furthermore, households that visited health facilities lacking sufficient and necessary laboratory services were twice as likely to drop out compared to those that accessed well-equipped facilities. This is likely because clients expect to receive appropriate treatment based on laboratory results. If referred elsewhere for diagnostic services, they may feel dissatisfied and become less inclined to renew their membership.

### Limitation of the study

As this study employed a cross-sectional design, it is subject to the inherent limitations of this methodology, particularly the inability to establish causal relationships between variables. Additionally, the exclusive use of a quantitative approach limits the depth of insight into community perceptions and contextual factors influencing CBHI dropout. A mixed-methods design incorporating qualitative components such as focus group discussions or key informant interviews would have provided a more comprehensive understanding of the underlying reasons for membership discontinuation.

## Conclusion and recommendation

### Conclusion

This study revealed a substantial dropout rate (36.1%) from the Community-Based Health Insurance (CBHI) scheme among rural households in Seyo district, Kellem Wollega Zone, Oromia, and Ethiopia. Several factors were significantly associated with the CBHI dropout rate, including a smaller family size, lower educational attainment, younger age of household heads, payer membership status, lack of chronic illness, poor experience at public health facilities, and limited access to necessary laboratory services. Therefore, we strongly recommend that the government and other stakeholders focus on identifying factors that reduce the dropout rate.

### Recommendations

Based on these findings, the following recommendations were forwarded to the concerned bodies.

For HEWs and kebele leaders

- Disseminating information on the CBHI scheme in a timely manner for all households in the kebele.
- Preparing community dialogue and briefing rumors regarding CBHI in the community

District and Zonal Health Office

- Availing all necessary services, like medications, at a health facility.
- Providing necessary and sufficient laboratory services at the health facility level
- Improving the delivery and quality of healthcare services to enhance CBHI members' health outcomes.
- Introducing flexible payment plans and reducing costs to make CBHI more affordable.

Regional Health Bureau and Federal Ministry of Health

- Organizing an advocacy meeting to raise awareness about the rationale behind the Community-Based Health Insurance (CBHI) scheme, aiming to reduce the high dropout rate.
- Strengthening supportive supervision and monitoring mechanisms to ensure the effective implementation and sustainability of the CBHI scheme.

## Author's note

Our current study employed a purely quantitative approach and recommended that future research adopt a mixed-methods design to better explore community perceptions and contextual factors related to the CBHI scheme.

## Data Availability

The original contributions presented in the study are included in the article/supplementary material, further inquiries can be directed to the corresponding author.
